# Relationship between extreme precipitation and acute gastrointestinal illness in Toronto, Ontario, 2012–2022

**DOI:** 10.1017/S0950268824000207

**Published:** 2024-02-08

**Authors:** Crystal J. Ethan, Johanna Sanchez, Lauren Grant, Jordan Tustin, Ian Young

**Affiliations:** 1 School of Occupational and Public Health, Toronto Metropolitan University, Toronto, Ontario, Canada; 2 Department of Population Medicine, University of Guelph, Guelph, Ontario, Canada

**Keywords:** acute gastrointestinal illnesses, climate change, flooding, precipitation, waterborne diseases

## Abstract

Extreme precipitation events are occurring more intensely in Canada. This can contaminate water sources with enteric pathogens, potentially increasing the risk of acute gastrointestinal illness. This study aimed to investigate the relationship between extreme precipitation and emergency department (ED) visits for acute gastrointestinal illness in Toronto from 2012 to 2022. Distributed lag non-linear models were constructed on ED visit counts with a Quasi Poisson distribution. Extreme precipitation was modelled as a 21-day lag variable, with a linear relationship assumed at levels ≧95th percentile. Separate models were also conducted on season-specific data sets. Daily precipitation and gastrointestinal illness ED visits ranged between 0 to 126 mm, and 12 to 180 visits respectively. Overall, a 10-mm increase in precipitation >95th percentile had no significant relationship with the risk of ED visits. However, stratification by seasons revealed significant relationships during spring (lags 1–19, peak at lag 14 RR = 1.04; 95% CI: 1.03, 1.06); the overall cumulative effect across the 21-day lag was also significant (RR = 1.94; 95% CI: 1.47, 2.57). Extreme precipitation has a seasonal effect on gastrointestinal health outcomes in Toronto city, suggesting varying levels of enteric pathogen exposures through drinking water or other environmental pathway during different seasons.

## Introduction

As a result of climate change, heavy precipitation events are occurring more frequently and intensely in many parts of the world. This has the potential to increase runoff, especially in areas that are mostly wet or frequently dry [1]. Heavy precipitation and consequently increased water runoff may introduce pathogens to water bodies and individuals are exposed to these pathogens through direct contact with contaminated water or food [2, 3]. Surface waters are vulnerable to microbial contamination since they lack any natural protection and filtration [4, 5]. As such, the microbial load of surface water may substantially increase after a precipitation event as a result of wash off from nearby agricultural lands, urban areas, and sewer discharges [3, 4, 6]. Additionally, increased overland runoff (due to heavy precipitation) can transport various pathogens from environmental reservoirs (e.g., animal manures, human sewage) into surface water. These events can result in contamination of surface water with various enteric pathogens, including toxin-producing *Escherichia coli, Campylobacter*, *Cryptosporidium,* and *Giardia* [7–9].

Several studies have revealed the impact of extreme precipitation on drinking water in Canada [7, 10–12]. For instance, Chhetri et al. found a significant increase in acute gastrointestinal illness (AGI) due to cryptosporidiosis and giardiasis 4–6 weeks after extreme precipitation events, with extreme precipitation having greater effects on AGI following a dry period [11]. Another study found an increased risk for AGI between 1 and 5 weeks after a heavy precipitation event [12]. However, of the studies executed so far, none took into consideration the explicit effects of other flooding indicators (stream discharge, sewer backup), especially indicators related to direct flooding exposures in homes. For instance, Chhetri et al.’s study in Vancouver investigated the effects of only extreme precipitation on the increase of cryptosporidiosis and giardiasis cases among residents [13]. Similarly, studies in Toronto (Ontario) also focused mainly on the effects of extreme precipitation only [2, 12]; one study investigated the turbidity of surface water with regards to heavy rainfall but did not consider exposure of turbid water exposure in residences [14]. With Toronto still using the combined sewer systems (which is prone to backing up/flowing out in homes), investigating the impacts of other flood indicators, especially those related to exposures in homes, is crucial [15]. Therefore, this study aims to examine the impact of extreme hydrological weather events on AGI outcomes in Toronto over a period of 10 years. The sub-objectives of the study include establishing a possible association between extreme precipitation (primary indicator of flooding) and AGI outcome, investigating the explicit effects of stream discharge and sewer backup (secondary flooding indicators) on AGI outcomes, and determining a relationship between water discoloration (indicator of water contamination) and AGI outcomes in Toronto city.

## Methods

### Study area

We conducted a time series analysis of the association between daily precipitation and daily counts of AGI ED visits in Toronto, Ontario, Canada, over a 10-year period, from 1 April 2012 through 31 March 2022. The City of Toronto has a population of over 3 million residents, over a land area of 630 km^2^ [16]. We hypothesized that heavy precipitation was associated with increases in AGI in the days after a heavy precipitation event. The City of Toronto gets its drinking water from Lake Ontario; water from the lake is treated (at the F.J. Horgan, Island Water, R.C. Harris, and R.L. Clark water treatment plants) and distributed to residents and businesses in Toronto and portions of York Region [17]. In addition to a few other bodies of water flowing into Lake Ontario, the four major rivers in Toronto (Etobicoke Creek, Rouge River, Humber River, and Don River) contribute immensely to the lake’s source [17]. A map of the administrative boundary for the City of Toronto is provided in Supplementary Figure S1, which shows the area of focus for our study as well as key environmental weather station locations and water treatment plant locations. A layer for the major Toronto rivers was added from the Toronto and Region Conservation Authority (Supplementary Figure S1).

### Data sets

Based on previous literature, AGI cases in this study were established as counts of all ED visits for AGI with the following ICD-10 codes that could be related to water exposures: A02 (salmonellosis), A03 (shigellosis), A04 (other bacterial intestinal infections), A05 (other bacterial foodborne intoxications, not elsewhere classified), A07.1 (giardiasis), A07.2 (cryptosporidiosis), A08 (viral and other specified intestinal infections), A09 (other gastroenteritis and colitis of infectious and unspecified origin), A32 (listeriosis), and B15 (acute hepatitis A) [18–20]. The AGI (outcome) data were obtained from the National Ambulatory Care Reporting System (NACRS) and covered 14 hospitals (with available data) across various areas in the City of Toronto [21].

The environmental data (predictors) were collected from several sources within and surrounding the city. Daily total precipitation (mm) and mean air temperature (°C) were obtained from Environment and Climate Change Canada weather stations [22]. For statistical purposes, the daily total precipitation (mm) was used to develop the extreme precipitation variable. An extreme/heavy precipitation event was defined as daily total precipitation greater than the 95th percentile of precipitation on all days during the study period. Data from Pearson airport station were used as the primary data set. Missing data were replaced with data from the Toronto Island station; a handful of remaining missing values from Pearson and Toronto Island stations were replaced with data from North York station, and one final missing value was inputted with data from the Toronto Buttonville airport station. Daily records from each of these stations were highly correlated, supporting our approach of filling in missing values from nearby stations where necessary. Stream flow (m^3^/s) data were extracted for the four major city rivers (Etobicoke Creek, Don River, Rouge River, and Humber River) from the Environment and Natural Resources Canada Historical Hydrometric archive [23]. These data were only available until the end of 2021. Data on the number of resident calls to the City of Toronto’s 311 service request system were obtained from Toronto Open Data for the call categories of ‘water discoloration (rusty/dirty)’ and ‘sewer main backups’ [24]. The 311 service request system provides residents, businesses, and visitors of Toronto with easy access to non-emergency city services, programmes, and information; calls made to 311 ranges from making reports of unleashed dogs, or dead animals, busted pipes, flooding in homes or on streets, illegal snow dumping, and much more [24]. For our study, the 311 data obtained were for reports of water discoloration and sewer backups in residences.

### Statistical analysis

An ecological, time series study design was employed to evaluate the association between extreme precipitation events and ED visits for AGI in the City of Toronto over the study period. We constructed distributed lag non-linear models (DLNM) with an overdispersed (quasi) Poisson distribution to investigate the dynamics of the association of interest [25]. First, we used DAGitty to create an initial directed acyclic graph (i.e., causal model) and establish the relationships (visual) between the exposure and outcome of interest, as well as potential confounding variables (identified through the review of previous similar studies) (Supplementary Figure S2) [26, 27]. In order for predictors (main and secondary) to be included in the model formula and to fit the DLNM requirements, cross-basis functions were constructed for each predictor variable (precipitation, stream discharge, water discoloration, sewer backup). In creating the cross-basis, we explored the linear, strata, and threshold functions and lags of 7, 14, and 21 days. These lags were selected with the consideration that the causative agents related to the onset of AGI typically have incubation periods between 1 and 7 days, except protozoa which can have longer incubation period (2–14 days) [2, 28, 29]. Additionally, AGI patients typically visit the emergency department (ED) after having symptoms for an average of 2–3 days; as such, these selected lag days were suitable to cover the duration from the onset of the illness to the time a clinical visit was made [2]. Furthermore, some previous literature have revealed significant associations between AGI and heavy precipitation about 1–6 weeks after a precipitation event [2, 3, 11, 12, 30]. Using the cross-basis, we then developed several base models (with precipitation and other covariates in separate models and combined into one model) to determine the suitable functional forms as well as the lag function for the non-linear relationship between the precipitation and AGI ED visits. Using the quasi Akaike’s Information Criterion goodness-of-fit statistic, we determined that the model with precipitation combined with other covariates, using the threshold function and lag 21, was the best fitted model for our data [31, 32]. This model structure was used for all of our models.

The primary exposure of interest was extreme precipitation (mm of rainfall). We modelled this variable as daily total precipitation with a threshold relationship at values ≥95th percentile (using df = 5, lag 21 for the cross-basis). The model was conditioned on the following covariates: air temperature, antecedent dry period, day of the week, season, COVID-19 pandemic period, and holidays. These variables were selected based on previous literature, as well as our understanding of certain variables (e.g., the pandemic lockdown) that could have some effect. Air temperature (°C) was evaluated as the daily mean for the day of the AGI ED visit. Antecedent dry period referred to the number of days with 0 mm of rainfall in the prior 30 days. This variable was developed based on the rationale that precipitation after a period of dryness will not be absorbed quickly by the soil, leading to more runoff water and contaminants, as well as a higher chance of contaminating water sources [10–13]. Seasons were defined as follows: winter (December, January, February), spring (March to May), summer (June to August), and fall (September to November). The pandemic period was considered to be all dates on or after the onset of the first provincial emergency declaration in Ontario (March 17, 2020). Long-term time trends were accounted for through a function of calendar time; using a natural spline model with 7 df per year; this and other dfs used for analysis were selected based on suggestions from Gasparrini and others, as well as those that were best fitted from our pre-analysis [25, 32].

We evaluated three separate models to examine alternative extreme weather variables: the effect of streamflow, service call volume for water discoloration, and service call volume for sewage backups. The streamflow variable (m^3^/s) represented the average daily discharge rate across the four major Toronto rivers (Etobicoke Creek, Rouge River, Humber River, and Don River). It was modelled as a threshold relationship at values ≥90th percentile (using df = 5, lag 21 for the cross-basis). This model had a better fit with the data than when the cut-off was set at the 95^th^ percentile. The water discolouration variable was an indicator of water treatment issues (turbidity), defined as the daily number of reports of dirty/rusty water by city residents (using df = 5, lag 21 for the cross-basis). Similarly, the sewage backup variable represented possible flooding and exposures at home (e.g., direct contact with sewage backup or flood water). It was defined as the daily number of reports of sewer main backups (using df = 5, lag 21 for the cross-basis). The water discolouration and sewage backup variables were both modelled as a threshold relationship at values above the minimum number of events (which was 0 for both variables). All secondary models adjusted for precipitation (using the same cross-basis initially constructed) and the same confounding variables as the primary model.

For both primary and secondary models, the analysis was first conducted on the overall data set and time period. In addition, the data were stratified into seasons and the analysis was repeated for each season. However, the df (for constructing the cross-basis) was decreased to 3 for seasonal analysis. As a sensitivity analysis, we examined the effects of precipitation on AGI at a threshold of the 90^th^ percentile of precipitation, while adjusting for the same covariates as the primary analysis. The same threshold was also applied to the secondary analyses (i.e., adjusting for precipitation at the 90^th^ percentile). Although we added a variable (pandemic) to adjust for the effect of the COVID-19 pandemic period in the primary analysis, we also conducted a sensitivity analysis, in which we excluded data covering the pandemic period (from 17 March 2020 onwards) to assess the robustness of the results. All sensitivity analyses was repeated for seasonal data as well. For all models, the percentage increase of relative risk (RR) was estimated as follows:

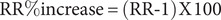



We defined a statistically significant relationship as one with a RR in which the 95% confidence interval excluded the null and had a *P* value that is <0.05. Also, we defined the first peak as the first RR > 1.00 over the 21-day lag period and the highest peak as the highest RR over the 21-day lag period. All analyses were conducted in R, version 4.2.3 (Shortstop Beagle) using dlnm version 2.4.7 [33–36].

## Results

**Figure 1.**
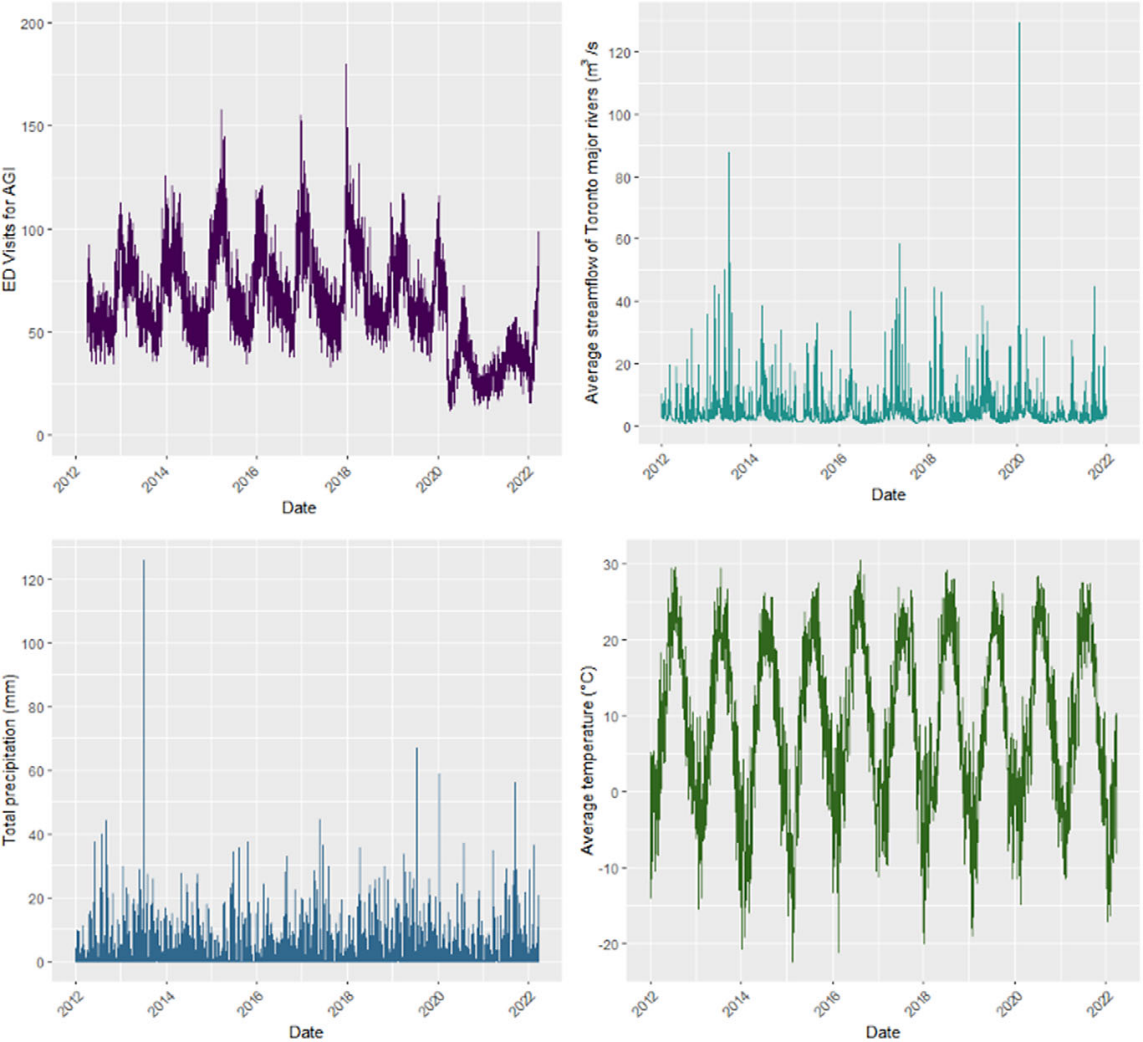
Timeseries plot of daily values during the study period, for: (a) counts of AGI emergency visits; (b) precipitation measurements; (c) average stream discharge of four major rivers in Toronto; (d) average daily air temperature.

A total of 230,663 ED AGI-related visits were reported in the City of Toronto between 1 April 2012 through 31 March 2022, ranging between 12 and 180 visits per day, with an average of 63 (Table 1). Although fall had the highest total count of AGI cases (68,381), winter recorded the highest daily AGI count (180), while summer noted the lowest total (47,594) and lowest daily (101) AGI counts during the study period. ED visits for AGI showed a clear seasonal/annual pattern (cases were high at the start and end of the year and low in between). There was a precipitation event (>0 mm) on 1,551 days (approximately 41.4%) of the study period. Daily precipitation ranged between 0 and 126 mm; the 90^th^ and 95^th^ percentiles of precipitation were 7.2 and 12.8 mm, respectively. The daily average stream flow ranged between 0.7 and 120 m^3^/s, while the 90^th^ and 95^th^ percentiles of stream flow were 8.5 and 12.4 m^3^/s, respectively. The full descriptive summaries and patterns for these and other predictors are presented in Table 1, Figure 1, and Supplementary Figure S3.

**Table 1.** Daily distributions of AGI visits and environmental variables during the study period, 2012–2022

	Mean (SD)	0p	50p	90p	95p	100p	Total count/days
Overall							
AGI cases	63.2 (23.1)	12.0	61.0	94.0	104.0	180.0	230,663
Precip (mm)	2.2 (5.7)	0.0	0.0	7.2	12.8	126.0	1,551
SD (m^3^/s)	4.1 (5.3)	0.7	2.5	8.5	12.4	120.0	3,653
Temp(°C)	8.9 (10.8)	−22.3	8.7	23.0	24.5	30.5	3,743
WD	0.9 (1.7)	0.0	0.0	2.0	3.0	45.0	3,161
SB	0.6 (1.5)	0.0	0.0	2.0	2.0	48.0	2,077
Winter							
AGI cases	70.1 (25.9)	13.0	80.0	117.0	115.0	180.0	63,197
Precip (mm)	1.9 (4.4)	0.0	0.0	5.6	10.8	59.0	470
SD (m^3^/s)	3.7 (5.2)	1.0	2.5	7.0	10.4	120.0	903
Temp (°C)	−1.5 (6.5)	−22.3	−0.8	6.2	8.8	16.1	962
WD	0.8 (1.3)	0.0	0.0	2.0	3.0	16.0	812
SB	0.5 (0.8)	0.0	0.0	1.0	2.0	5.0	484
Spring							
AGI cases	70.0 (25.9)	12.0	73.0	100.0	108.0	158.0	51,491
Precip (mm)	2.1 (4.9)	0.0	0.0	6.8	12.0	44.6	382
SD (m/s^3^)	5.4 (5.9)	1.0	3.4	11.6	17.6	52.4	920
Temp (°C)	4.3 (8.8)	−22.3	4.3	15.8	18.6	25.8	951
WD	0.9 (1.4)	0.0	1.0	2.0	3.0	19.0	757
SB	0.5 (0.9)	0.0	0.0	1.0	2.0	17.0	443
Summer							
AGI cases	55.9 (12.0)	23.0	57.0	71.0	75.0	101.0	47,594
Precip (mm)	2.3 (7.0)	0.0	0.0	7.8	15.1	126.0	319
SD (m^3^/s)	3.7 (4.8)	0.7	2.3	7.4	10.7	74.5	920
Temp (°C)	19.6 (4.9)	0.9	20.3	25.3	26.5	30.5	920
WD	1.0 (2.2)	0.0	1.0	3.0	3.0	45.0	895
SB	0.8 (2.4)	0.0	0.0	3.0	3.0	48.0	667
Fall							
AGI cases	51.7 (13.4)	15.0	52.0	67.1	72.0	111.0	68,381
Precip (mm)	2.2 (5.5)	0.0	0.0	7.8	13.4	56.0	380
SD (m^3^/s)	3.1 (3.6)	0.7	2.0	6.2	9.5	52.6	910
Temp (°C)	13.6 (8.0)	−9.6	14.9	23.4	24.9	30.5	910
WD	0.9 (1.2)	0.0	0.0	2.0	3.0	9.0	697
SB	0.6 (1.0)	0.0	0.0	2.0	2.2	9.0	483

*Note:* Total count reports the sum of occurrences (AGI cases, water discoloration events, and sewer backup events), and total days reports the number of days an event occurred or was reported (precipitation, stream discharge, and air temperature).

Abbreviations: AGI, acute gastrointestinal illness; p, percentile; Precip, precipitation; SB, sewer backup (events of sewer backup in homes); SD, stream discharge/flow; Temp, air temperature; WD, water discoloration (events of water discoloration in homes).

Table 2 shows a summary of associations between environmental variables and AGI incidence in Toronto. In the overall analysis, extreme precipitation was not statistically associated with AGI-related ED visits; the association rather showed a trend towards a protective effect. However, the seasonal analysis revealed that extreme precipitation during the spring season was statistically associated with an increase in visits for AGI. For every 10 mm increase above the 95^th^ percentile (12.8 mm) of precipitation during spring, there was a 94.3% increase in RR for Toronto residents to visit an ED for AGI. Given the RR for the relationship between extreme precipitation events and AGI during the summer and fall seasons, we consider heavy rainfall to be marginally associated with an increase in AGI illnesses in Toronto in the summer and fall seasons, in addition to the spring season. Similar to precipitation, stream discharge (overall) did not have a significant relationship with AGI incidence but showed a trend towards a protective effect. Stratification by season revealed marginal positive associations between stream discharge and ED visits for AGI in summer and fall (Table 2).

**Table 2.** RR associated with extreme precipitation, individual flooding indicators, and AGI emergency department visits in Toronto, 2012 to 2022

	RR (95% CI)	RR% increase	RR (95% CI)	RR% increase
	Precipitation		Stream discharge	
Overall	0.92 (0.83, 1.01)	−8.0	0.97 (0.92, 1.02)	−3.0
Winter	1.02 (0.69, 1.51)	2.0	1.21 (0.99, 1.47)	21.0
Spring	1.94 (1.47, 2.57)^a^	94.0	0.95 (0.85, 1.05)	−5.0
Summer	1.07 (0.95, 1.19)	7.0	1.15 (0.95, 1.40)	15.0
Fall	1.38 (1.10, 1.73)	38.0	1.22 (0.91, 1.62)	22.0
	Water discoloration		Sewage backup	
Overall	0.88 (0.75, 1.02)	−12.0	1.06 (0.87, 1.28)	6.0
Winter	0.67 (0.45, 1.02)	−33.0	2.91 (1.55, 5.46)^a^	191.0
Spring	0.63 (0.43, 0.91)	−38.0	13.06 (7.83, 21.77)^a^	1,206.0
Summer	1.16 (1.00, 1.39)^a^	16.0	1.02 (0.82, 1.28)	2.0
Fall	1.87 (1.12, 3.15)	87.0	1.14 (0.67, 1.91)	14.0

Abbreviations: AGI, acute gastrointestinal illness; CI, confidence interval; RR, relative risk.

a Statistically significant.

The number of service calls related to the occurrence of discoloured water in homes was significantly associated with an increased ED visits for AGI during the summer; every 10 calls related to water discoloration was associated with a 16% increase in AGI risk. Also, a trend towards a significant association was observed for events of water discoloration during the fall season, with a high RR [1.87 (1.12, 3.15)] of ED visits for AGI. The number of service calls related to sewage backup events posed the highest increased risk of developing AGI in Toronto. Particularly, seasonal observations revealed a very high percentage increase in RR during spring and winter seasons. In the sensitivity analysis (i.e., precipitation at the 90^th^ percentile), only events of sewage backup during winter and spring remained significantly associated with AGI incidence in Toronto (Supplementary Table S1).

**Figure 2.**
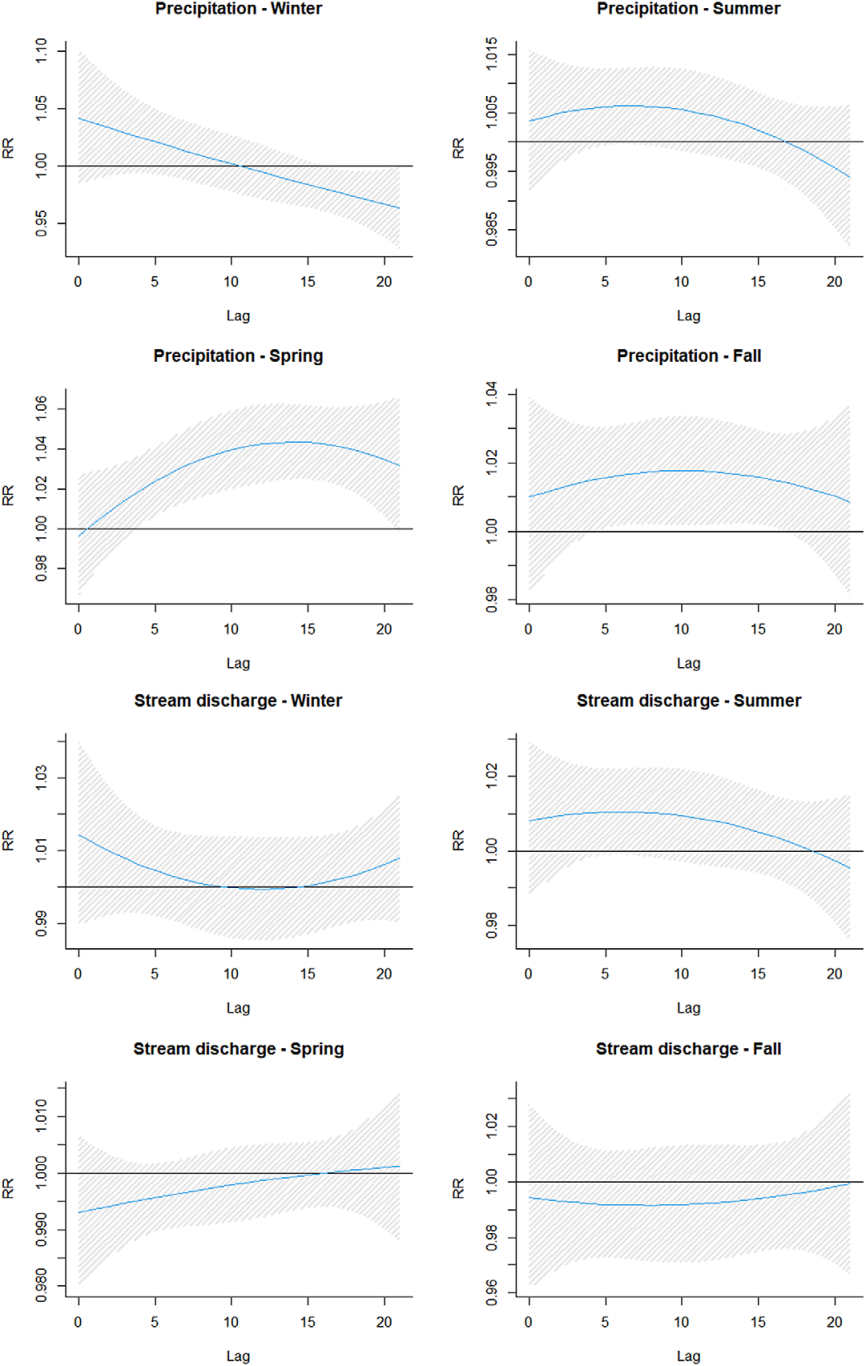
Graphical presentation of the association between extreme precipitation, stream discharge and AGI incidence in Toronto across a 21-day lag.

Figure 2 and Table 3 show the delayed effects of extreme precipitation and other flooding indicators on ED AGI visits in Toronto between 2012 and 2022. The RRs given report the first peak of an increased rate across lag days (we highlighted the first peak because some associations peaked a second time over the 21-day lag period) and the highest peak. The first peak gives clarity as to how long the effects of precipitation and other predictors lingered before a positive association (increased risk) with AGI was established. In some cases, as seen with overall precipitation and water discoloration during winter and spring, there were no positive associations over the entire 21-day lag (all RR were < 1.00) (Table 1, Figure 2, Supplementary Figure S4). However, stratification by seasons revealed that the increased risk of AGI outcome was established immediately after an extreme precipitation event during winter, summer, and fall seasons and 1 day after an extreme precipitation event during spring. With the exception of the winter season, the risk of AGI after the first peak continued to increase and only declined around lag 17 (Figure 2). On the other hand, stream discharge (in spring) and events of sewage backup (in fall) did not suggest an immediate effect on risk for AGI; increased risk was established 18 and 8 days, respectively, after exposure. Associations with delayed first peaks also noted second peaks, most towards the end of the 21-day lag period.

**Table 3.** Associations of extreme precipitation, flooding indicators, and AGI cases at the first and highest lag with RR > 1

	Precipitation					
Season	Lag with first peak >1	RR (95%CI)	RR% increase	Lag with highest peak	RR (95%CI)	RR% increase
Overall	-	-	-	9	1.00 (0.99, 1.01)^a^	−0.0
Winter	0	1.04 (0.99, 1.10)	4.0	0	1.04 (0.99, 1.10)	4.0
Spring	1	1.00 (0.98, 1.03)^a^	0.0	14	1.04 (1.03, 1.06)	4.0
Summer	0	1.00 (0.99, 1.02)^a^	0.0	7	1.00 (1.00, 1.01)	0.0
Fall	0	1.01 (0.98, 1.04)	1.0	10	1.02 (1.0, 1.03)	2.0
	Stream discharge					
Overall	0	1.01 (1.00, 1.02)	1.0	0	1.01 (1.00, 1.02)	1.0
Winter	0	1.02 (1.00, 1.05)	2.0	0	1.02 (1.00, 1.05)	2.0
Spring	18	1.00 (0.99, 1.01)^a^	0.0^a^	21	1.00 (0.99, 1.01)^a^	0.0
Summer	0	1.01 (0.99, 1.03)	1.0	6	1.01 (1.00, 1.02)	1.0
Fall	0	1.01 (0.98, 1.04)	1.0	10	1.01 (0.99, 1.03)	1.0
	Water discoloration					
Overall	9	1.00 (0.99, 1.01)^a^	0.1	11	1.00 (0.99, 1.02)^a^	0.0
Winter	-	-	-	12	0.97 (0.96, 1.02)	−3.0
Spring	-	-	-	12	0.99 (0.96, 1.01)	−1.0
Summer	4	1.00 (0.992, 1.014)^a^	0.3	12	1.02 (1.01, 1.03)	2.0
Fall	0	1.03 (0.97, 1.09)	3.0	21	1.04 (0.98, 1.11)	4.0
	Sewer main backup					
Overall	0	1.01 (0.99, 1.04)	1.0	0	1.01 (0.96, 1.04)	1.0
Winter	4	1.01 (0.96, 1.06)	1.0	12	1.12 (1.07, 1.17)	12.0
Spring	0	1.03 (0.96, 1.11)	3.3	21	1.18 (1.09, 1.29)	18.0
Summer	0	1.01 (0.98, 1.03)	1.0	0	1.01 (0.98, 1.03)	0.6
Fall	8	1.00 (0.96, 1.04)^a^	0.0	19	1.04 (0.99, 1.10)	4.0

Abbreviations: AGI, acute gastrointestinal illness; RR, relative risk.

a Indicates the RR and RR% increase are >1 when the value has not been rounded up.

The highest peak for extreme precipitation was observed during spring at lag 14; every 10 mm increase above the 95^th^ percentile of precipitation was associated with 4.4% increased risk of AGI. Events of water discoloration and sewage backup noted their highest peaks at lag 21 during fall and spring, respectively. Both predictors also noted the longest lag span (lag 0–21) with positive associations with AGI emergency visits; the risk percentage increase ranged between 2.8% and 4.2% and 3.3% and 18.3%, respectively (Table 3, Supplementary Figure S4). Unlike other associations, the delayed effect of stream discharge on AGI risk was highest at its first peak, lag 0, during winter but subsequently decreased over the 21-day lag period (Figure 2). However, the longest lag span during which stream discharge was positively associated with AGI risk was lag 0–10 in the fall season, with risk percentage ranging between 0.8% and 1.0%.

Substantial differences were not observed between the lagged effects for the primary and sensitivity analyses. A few notable changes included a quicker lag-response relationship for stream discharge and AGI during spring. The first and highest peaks were at lag 18 and 21, respectively, for the primary model; however, for the sensitivity model, observations were at lag 10 and 15, respectively. Nonetheless, the RRs were similar and not statistically significant in both models. The opposite was noted for the sewage backup variable; its highest peak during summer was observed at lag 0 in the primary model and at lag 4 in the sensitivity model. Results from the COVID-19 sensitivity analysis were slightly lower than that of the primary analysis. However, the statistical significance for both analyses were the same (overall and per season) (Supplementary Table S3).

## Discussion

Epidemiological research undertaken in high-income nations like the United States, Canada, Taiwan, France, England, and Australia have shown a link between heavy precipitation and an increased risk of AGI [3, 4, 7, 10, 35, 36]. This association raises a significant public health concern since it may indicate microbial contamination of drinking water supplies that have evaded treatments by water systems or could indicate exposure from direct contact with floodwater or residential sewage backups resulting from heavy rainfall events. Depending on the source waters for drinking water supplies, upstream land use, impermeable land cover, and the presence of combined sewer systems, certain countries and regions are more exposed than others to health-related issues from heavy precipitation events [30]. During heavy rains, water treatment plants may become overburdened, resulting in possible cross-contamination between sewage and drinking water pipes, sewage overflow, or bypasses into local waterways, residences, or other properties [6]. This occurs mostly where older water infrastructures (such as combined sewer systems) are used. Where in use, the combined sewers carry all contents (rain, melted snow, and sewage) simultaneously, and consequently combined sewer overflows contain various pathogens, heavy metals, oils, pesticides, and nutrients that can increase microbial growth and compromise the quality of water [6, 15]. Additionally, exposure to faecal contamination is more likely, especially in areas with combined sewer systems. A combined sewer system is still used by a lot of Toronto residents, which increases the likelihood of a sewer flooding event because combined sewers overflow more frequently during heavy rain than separate sewers do. The percentage of residents using the combined sewer system varies across provinces; for example, 21% and 44% of residents of Quebec and Vancouver, respectively, use the combined sewer systems [37, 38].

In Canada, waterborne enteric illnesses remain a significant public health concern despite ongoing developments in water treatment infrastructure and analytical monitoring methods [39]. There’s an estimated 20.5 million AGI cases in Canada annually; of these, 334,966 cases are estimated to be associated with the consumption of tap water from municipal systems (Canadian municipal drinking water systems) [40]. With 83% of Canada’s population consuming tap water from municipal drinking water plants, these estimates (associated with tap water consumption) might be higher given that most cases are under-diagnosed and under-reported [40, 41].

Using several sources of data, our study examined the lagged relationship between extreme precipitation and emergency visits for AGI in the City of Toronto over a 10-year period. Our hypothesis was that any associated increase in AGI risks was most likely to occur via waterborne transmission, but indirect contamination through contact with contaminated floodwater was also considered as a possibility. We found a significant association between extreme rainfall and ED visits for AGI during spring only, with a cumulative increased risk of 94.3% over a 21-day lag. The exclusion of the pandemic period had no significant effect on the primary findings of the study. Overall precipitation was still not statistically associated with AGI incidence in Toronto, and similar to the primary results, only the stratification by seasons revealed significant association between the two variables (precipitation and AGI) in spring season. A minor impact (slightly lower RR) on the winter season results was noted but was insufficient to change or alter the primary findings. A mechanical pathway involving waterborne transmission of AGI starts with heavy precipitation resulting in higher than acceptable levels of pathogens entering source waters, either through stormwater runoff or from sewer overflows or the re-suspension of stream sediments [42]. Individuals could then be exposed by consuming contaminated water or having direct contact with river water, floodwater, or sewage backups. For instance, numerous studies have linked cryptosporidiosis (a frequent cause of waterborne gastroenteritis) to high rainfall, most likely as a result of runoff and flooding contaminating water sources [10, 11, 37, 43]. A study in New Jersey discovered that *Cryptosporidium* concentration rose after rainfall and was even more closely connected with other factors like streamflow and turbidity than rainfall [42].

Other than consumption of contaminated water, we considered whether contact with recreational waters may also have contributed to the increases in AGI cases. However, the plausibility of this remains questionable, considering that the relationship between precipitation and AGI was significant only during spring, when contact with recreational water is low due to the cold ambient and water (river/lake)/air temperatures. Contact with flood waters and sewer backups, especially in homes, are also plausible exposure routes through which individuals may come into contact with various enteric pathogens [44]. In Toronto, the city’s older areas use combined sewers in which only one pipe carries both sewage and stormwater. During periods of heavy rainfall, the volume of stormwater that enters these combined sewers may exceed the system’s capacity and overflow directly into water bodies (creeks, rivers, lakes) or flood private properties or public spaces (via catch basin backup or overflows) [15, 45].

Regardless of the exact route of transmission, the aetiology of AGI is due mostly to viral agents (mainly rotavirus, norovirus, and other enteric viruses), bacterial pathogens (such as *Campylobacter*, *Salmonella*, or toxin-producing *E. coli*), and/or protozoa (such as *Giardia* and *Cryptosporidium*). As at 2021, *Campylobacter, Cryptosporidium, Giardia, Salmonella, Shigella,* and Verotoxin-producing *E. coli* were identified as some of the most common pathogens causing foodborne/waterborne illnesses among Ontario residents [46–48]. These causative agents typically have an incubation period of 1 to 7 days, except protozoa which can have longer incubation periods (2–10 days and 1–14 days for *Cryptosporidium* and *Giardia*, respectively) [2, 28, 29]. We found a consistent positive association between extreme precipitation and AGI from 4 to 20 days following a heavy precipitation event during the spring season. The increased risk during this period ranged from 1.9% to 4.4% for every 10 mm above the 95^th^ percentile of precipitation (Table 2). The sensitivity analysis also revealed similar associations. Given that AGI patients tend to visit the ED after having symptoms for an average of 2–3 days [2], the observed time lag in our study is consistent with the expected timing of exposure.

We found a marginal association with stream discharge (the average volume of water flowing in the four major rivers in this study) and ED visits for AGI in Toronto (Table 2). As such, we consider its effect may be closely linked to the risk observed for extreme precipitation. Stream flow is affected by weather (increasing during rainstorms and decreasing during dry periods) and seasons (decreasing during summer when evaporation is high and shoreline vegetation is actively growing and removing water from the ground) [49]. We found a moderate correlation (*r* = 0.409) between stream discharge and precipitation, indicating the increase or decrease of both variables was moderately proportional. Such relationships are known to influence the kinds of organisms and the amount of silt and sediment found in water bodies [50, 51]. Sediments introduced to slow-flowing streams (indicative of low precipitation) will settle quickly to the stream bottom and reduces the chances of human contact. However, fast moving streams (indicative of high precipitation) will keep sediments suspended longer in the water column, increasing the chances of human contact [50,51]. These phenomena align with the findings of our study, given that stream flow was higher during spring (90^th^ percentile: 11.6 m^3^/s) than summer (90^th^ percentile: 7.4 m^3^/s) and only during spring did precipitation have a significant relationship with AGI (Table 1). Understanding the main and interactive effects of precipitation and stream flow on AGI incidence is needed to securely establish this finding.

While other studies mostly noted that the incidence of AGI is responsive to precipitation events, our study additionally examined indicators of water quality (reports of water discoloration) and exposure of flood water in and around residences (reports of sewage backups). Service calls related to water discoloration in Toronto were significantly associated with ED visits for AGI during the summer. Similar to precipitation, the lag span during which delayed effects were noted coincides with the incubation period of many causative agents of AGI. Water discoloration may be a consequence of inadequately treated water supplies, equipment or infrastructure repairs or malfunctions, construction, or other factors (e.g., nearby fire hydrant use) [11, 12]. Water colour is affected by a combination of inorganic and organic substances that, when present in the water at high enough quantities, produce excessive turbidity or colour that is perceptible to consumers [52]. Inorganic compounds of concern include iron and manganese that can originate from the source water and/or be released from the pipe wall due to corrosion reactions or hydraulic events [53]. Organic substances may be a result of heavy precipitation following long dry periods [54–56]. Prolonged dry spells cause the soil to become compact and retain contaminants, which when followed by an intense rainfall event, allows for overland runoff into sources of drinking water, increasing turbidity and pathogen loads [57–59]. Such events have the potential to overwhelm drinking water treatment processes, allowing pathogens to pass through to the distribution system and ultimately to residents’ homes where they are ingested [12, 60]. The findings of our study suggest this could be a possible route of exposure in Toronto, given that summer has relatively longer dry periods than other seasons.

Of all predictors observed in our study, service calls related to sewage backup in and around homes had the highest increased risk for AGI ED visits in Toronto. This may be related to the fact that sewer backups are strongly influenced by heavy precipitation (sewer backup was significantly associated with heavy precipitation) and also suggests the severity of exposure to sewer backup (which could make sewer backup an indicator of AGI risks in Toronto city). The association between events of sewer backup and AGI was significant in winter and spring seasons and remained robust for all sensitivity analyses and adjustments. Heavy precipitation causes sewer lines to backup into basements when municipal storm drains reach maximum capacity and the excess water flows into residential sewer lines. Although there are other causes of sewer backup, the significant association noted during seasons typical of heavy precipitation may indicate precipitation, in this case, primarily influenced the reported events [61, 62]. Such flooding events should be considered a serious health concern with regards to AGI risks, as this could lead to direct contact to enteric pathogens [10, 30, 43]. There is a high tendency of faecal contamination exposure, especially where a combined sewer system is used. About 23% of the City of Toronto still uses a combined sewer system, which has a much higher chance of experiencing a sewer flooding event, given that combined sewers tend to flood easily during heavy rain than separated sewers [52]. There are likely many under-reported incidents of sewer backup events. According to Toronto Water, for a single sewer backup event in a home reported to 311, there is a possibility that multiple properties on the same street may have experienced similar flooding (a likely indication that the sanitary sewer under the street has a blockage) [61]. For each report of a sewer backup, it is likely that several residents are affected. As a measure to combat sewer flooding, the City of Toronto is currently undertaking a comprehensive, multi-year, multi-billion dollar infrastructure plan to reduce the impact of combined sewers on water quality [15]. A major outline of the project is keeping combined sewer overflows out of key waterways and the harbour by capturing sewage within a tunnel system, storing it during heavy rain until system capacity is restored, and then transporting it for treatment. Additionally, a newly built integrated pumping station will move raw sewage from underground sewers into a treatment plant to provide additional capacity for outflow and replace two ageing pumping stations [63].

There are some limitations of this study. As we only had access to aggregated illness data, we could not stratify by gender and age to give more insights on specific populations and group risks. We also could not group AGI incidence into different categories based on ICD classifications, which could have revealed more specific associations with different aetiologies. Additionally, under-reporting of AGI cases is likely given that ED visits are mainly representative of more severe cases. Less severe cases of AGI which do not make it to the ED may have a different relationship with the predictors of interest. This is because less severe cases are often found in adults, while more severe cases that are reported/diagnosed are mostly found in children. As such, the data used may not be representative of all age categories. It is also important to note that the health data used did not reveal if AGI was or was not the primary illness or reason for the ED visit. As common with most ecological studies, exposure data were only available at the city level. The weather conditions and exposure levels observed in the study were based on city-wide data and not specific to each patient or their homes. This challenge may limit knowledge of the precise relationship between heavy precipitation and AGI, since weather conditions and exposure levels can vary from location to location. The streamflow and 311 call data were only available until the end of 2021; this may only minutely reduce the statistical power of the secondary analyses but will likely not have a large impact on the results due to the volume of data used. Also, the 311 call data (for occurrence of water discoloration and sewer backup) were more of syndromic surveillance indicator of a possible signal of issues or direct exposure to flooding at home and does not reflect precise measurement of these indicators. Additionally, since the 311 call data were self-reported secondary data, it was impossible to determine if the events reported by residents were accurate.

## Conclusion

As extreme weather events become more frequent due to climate change, it is important to identify their possible impacts on water systems and anticipate possible health risks. We found an association between heavy precipitation (in spring), sewer backup reports (in winter and spring), and water discoloration reports (in summer) and the number of ED visits for AGI in the City of Toronto. Overall, we found a seasonal effect of precipitation and other flood indicators on the health of Toronto residents, specifically AGI outcomes. Tailored monitoring, sampling, and additional research to determine specific pathogens and exposure pathways associated with each season may be warranted to ensure ideal water treatment and safety. Additionally, public health messaging and risk communication about the immediate and delayed effects of heavy precipitation and its related events on AGI should be considered to raise awareness of possible illness risks and risk mitigation strategies and to encourage reporting of flooding events (e.g., sewage backups) to support syndromic surveillance.

The use of 311 service for syndromic surveillance of AGI (or other waterborne/foodborne illnesses) should be explored to provide residents a plethora opportunities to report mild or severe symptoms prior to ensure all cases in the general public are captured.

## Supporting information

Ethan et al. supplementary material Ethan et al. supplementary material

## Data Availability

The environmental data sets analysed in this study are publicly available from the City of Toronto and Government of Canada open data sets, as referenced in the Methods section. The emergency department visit data were obtained from the Canadian Institute for Health Information. The final aggregated data set used in the analysis can be requested from the authors.

## Supplementary material

For supplementary material accompanying this paper visit http://doi.org/10.1017/S0950268824000207. click here to view supplementary material
