# Supplementary material for: Relationship between extreme precipitation and acute gastrointestinal illness in Toronto, Ontario, 2012–2022
Source: Epidemiol Infect. 2024 Feb 8;152:e32. doi: 10.1017/S0950268824000207 (PMC10894888; doi:10.1017/S0950268824000207)
Supplement: Ethan et al. supplementary material [file S0950268824000207sup001.docx]

Epidemiology and infection

Relationship between extreme precipitation and emergency department visits for acute gastrointestinal illness in Toronto, Ontario, 2012-2022.

Crystal J. Ethan, J. Johanna Sanchez, Lauren Grant, Jordan Tustin, Ian Young

# Supplementary Material

Supplementary Table S1: Relative risk associated with extreme precipitation, flooding indicators and AGI emergency department visits in Toronto, 2012 to 2022 (Sensitivity analysis).

|  | Precipitation | | Stream discharge | |
| --- | --- | --- | --- | --- |
|  | RR(95%CI) | RR% increase | RR(95%CI) | RR% increase |
| Overall | 0.93(0.86, 1.00) | -7.0 | 0.97(0.92, 1.03) | -3.0 |
| Winter | 0.97(0.738, 1.266) | -3.0 | 1.11(0.94, 1.32) | 11.0 |
| Spring | 1.40(1.135, 1.733) | 40.0 | 0.98(0.88, 1.10) | -2.0 |
| Summer | 1.07(0.973, 1.171) | 7.0 | 1.12(0.92, 1.37) | 12.0 |
| Fall | 1.20(1.01, 1.43) | 20.0 | 1.36(1.01, 1.82) | 36.0 |
|  | | | |  |
|  | Water discoloration | | Sewer-backup | |
| Overall | 0.87(0.75, 1.02) | -13.0 | 1.03(0.861, 1.227) | 3.0 |
| Winter | 0.71(0.47, 1.07) | -29.0 | 2.98(1.58, 5.64)* | 198.0 |
| Spring | 0.70(0.48, 1.02) | -30.0 | 13.83( 8.29, 23.07)* | 1,283.0 |
| Summer | 1.16(0.96, 1.38) | 16.0 | 0.99(0.81, 1.21) | -1.0 |
| Fall | 1.86(1.10 3.14) | 86.0 | 1.13(0.67 1.88) | 13.0 |

* statistically significant

Supplementary Table S2: Associations of extreme precipitation, flooding indicators and AGI cases at the first and highest lag with relative risk > 1.

|  | Precipitation | | | | | |
| --- | --- | --- | --- | --- | --- | --- |
| Season | Lag with first peak >1 | RR(95%CI) | RR% increase | Lag with highest peak | RR(95%CI | RR% increase |
| Overall | - | - | - | 10 | 1.00(0.99, 1.01) | 0.0 |
| Winter | 0 | 1.01(0.98, 1.04) | 1.0 | 4 | 1.01(0.99, 1.03) | 1.1 |
| Spring | 1 | 1.00(0.98, 1.02)+ | 0.0 | 15 | 1.02(1.01, 1.04) | 2.2 |
| Summer | 0 | 1.00(0.99, 1.01)+ | 0.0 | 7 | 1.01(1.00, 1.01) | 1.0 |
| Fall | 0 | 1.00(0.98, 1.02) | 0.0 | 14 | 1.01(1.00, 1.02) | 1.0 |
|  | | | | | | |
|  | Stream discharge | | | | | |
| Overall | 0 | 1.01(1.00, 1.02) | 1.0 | 0 | 1.01(1.00, 1.02) | 1.0 |
| Winter | 0 | 1.03(1.00, 1.05) | 3.0 | 0 | 1.03(1.00, 1.05) | 3.0 |
| Spring | 10 | 1.00(0.99, 1.01)^+^ | 0.0^+^ | 15 | 1.00(0.99, 1.01) | 0.0 |
| Summer | 0 | 1.02(0.99, 1.03) | 2.0 | 6 | 1.01(1.00, 1.02) | 1.0 |
| Fall | 0 | 1.02(0.99, 1.05) | 2.0 | 0 | 1.02(0.99, 1.05) | 2.1 |
|  | | | | | | |
|  | Water discoloration | | | | | |
| Overall | 9 | 1.00(0.99, 1.01)^+^ | 0.0^+^ | 11 | 1.00(0.99, 1.02) | 0.0 |
| Winter | - | - | - | 13 | 0.99(0.96, 1.02) | -1.0 |
| Spring | - | - | - | 10 | 1.00(0.97, 1.02) | -0.0 |
| Summer | 4 | 1.00(0.99, 1.01) | 0.0 | 12 | 1.02(1.01, 1.03) | 2.0 |
| Fall | 0 | 1.02(0.97, 1.08) | 2.0 | 21 | 1.04(0.98, 1.11) | 4.0 |
|  | | | | | | |
|  | Sewer main backup | | | | | |
| Overall | 0 | 1.01(0.99, 1.04) | 1.0 | 0 | 1.01(0.99, 1.04) | 1.0 |
| Winter | 4 | 1.01(0.97, 1.06) | 1.0 | 12 | 1.12(1.07, 1.17) | 12.0 |
| Spring | 0 | 1.04(0.97, 1.12) | 4.0 | 21 | 1.18(1.09, 1.29) | 18.0 |
| Summer | 0 | 1.00(0.97, 1.03)+ | 0.0 | 4 | 1.00(0.99, 1.02) | 0.0 |
| Fall | 8 | 1.00(0.97, 1.04)+ | 0.0 | 18 | 1.04(1.00, 1.09) | 4.0 |

+ indicates the RR and RR% increase are >1 when the value has not been rounded up.

Supplementary Table S3: Relative risk associated with extreme precipitation and AGI emergency department visits in Toronto, 2012 to 2022 (Sensitivity analysis excluding the pandemic data).

|  | All data | | | Excluding Covid period | | |
| --- | --- | --- | --- | --- | --- | --- |
|  | Precipitation at the 95^th^ percentile | | | | | |
|  | RR(95%CI) | RR% increase | | RR(95%CI) | RR% increase | |
| Overall | 0.92 (0.83, 1.01) | | -8.0 | 0.85 (0.760, 0.94) | | -15.0 |
| Winter | 1.02 (0.69, 1.51) | | 2.0 | 0.62(0.41, 0.93) | | -38.0 |
| Spring | 1.94 (1.47, 2.57)* | | 94.0 | 1.68(1.27, 2.21)* | | 68.0 |
| Summer | 1.07 (0.95, 1.19) | | 7.0 | 1.03(0.92, 1.14) | | 3.0 |
| Fall | 1.38 (1.10, 1.73) | | 38.0 | 0.88(0.67, 1.14) | | -12.0 |
|  | Precipitation at the 90^th^ percentile | | | | | |
| Overall | 0.93(0.86, 1.00) | | -7.0 | 0.88 (0.80, 0.96) | | -12.0 |
| Winter | 0.97(0.74, 1.27) | | -3.0 | 0.75(0.57, 0.98) | | -25.0 |
| Spring | 1.40(1.14, 1.73) | | 40.0 | 1.20(0.97, 1.48) | | 20.0 |
| Summer | 1.07(0.97, 1.17) | | 7.0 | 1.02(0.93, 1.12) | | 2.0 |
| Fall | 1.20(1.01, 1.43) | | 20.0 | 0.93(0.77, 1.13) | | -7.0 |

| 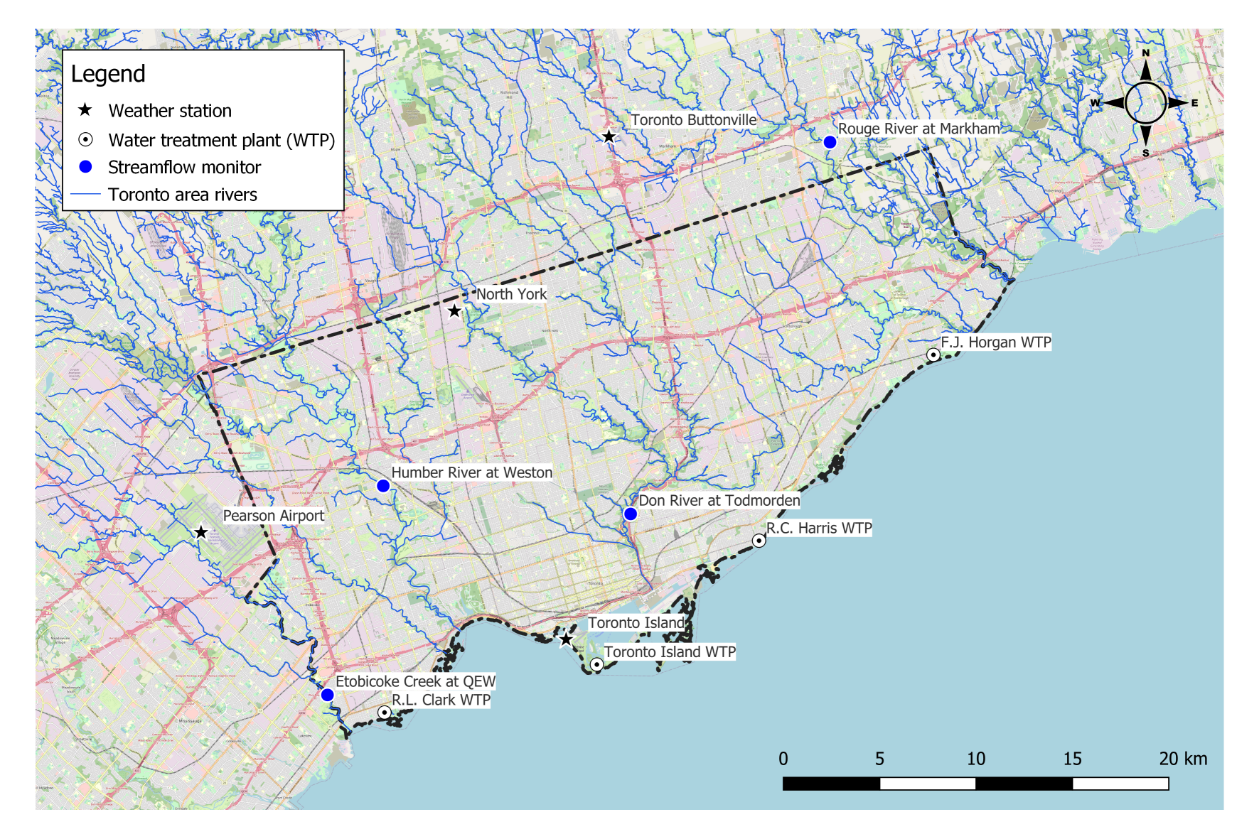 |
| --- |
| Supplementary Figure S1: The study region (Toronto city), showing the various weather stations, major rivers, water treatment plants, streamflow monitors and riverine areas. |

| 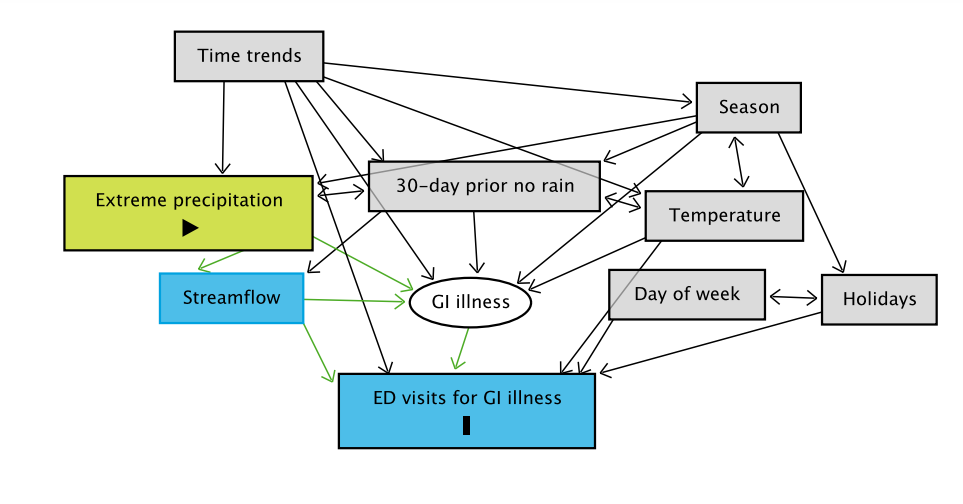 |
| --- |
| Supplementary Figure S2: Graphical presentation of the selection of predictors and covariates to be used in our models. |

| 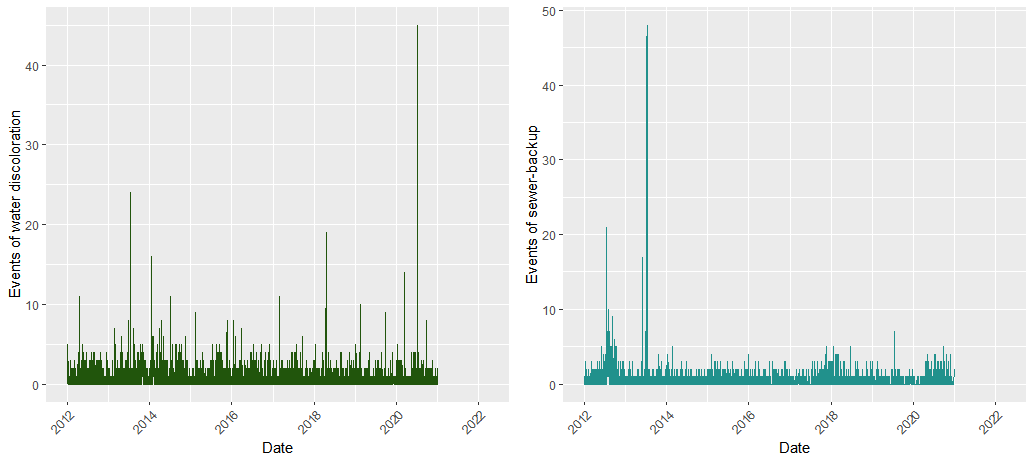 |
| --- |
| Supplementary Figure S3: Time-series plots of daily values during the study period, for: (a) events of water discoloration; (b) Events of sewer-backup. |

| 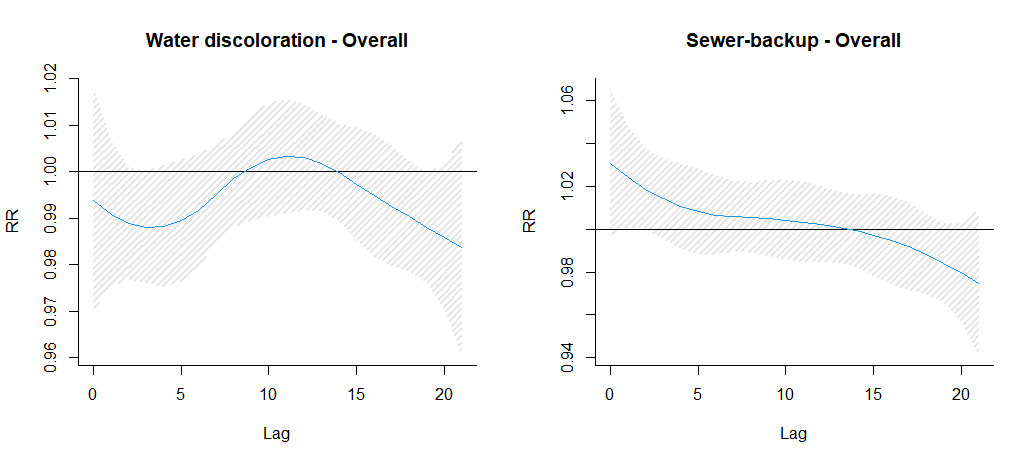 |
| --- |
| 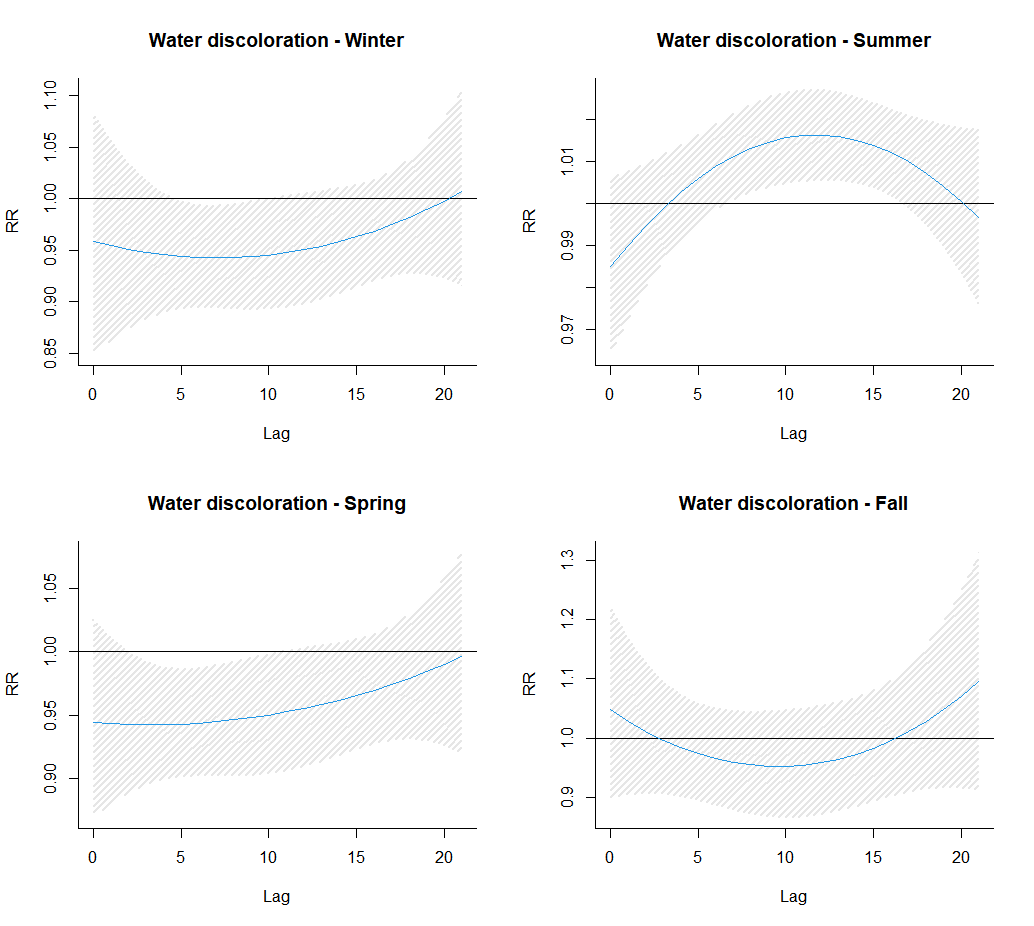 |
| 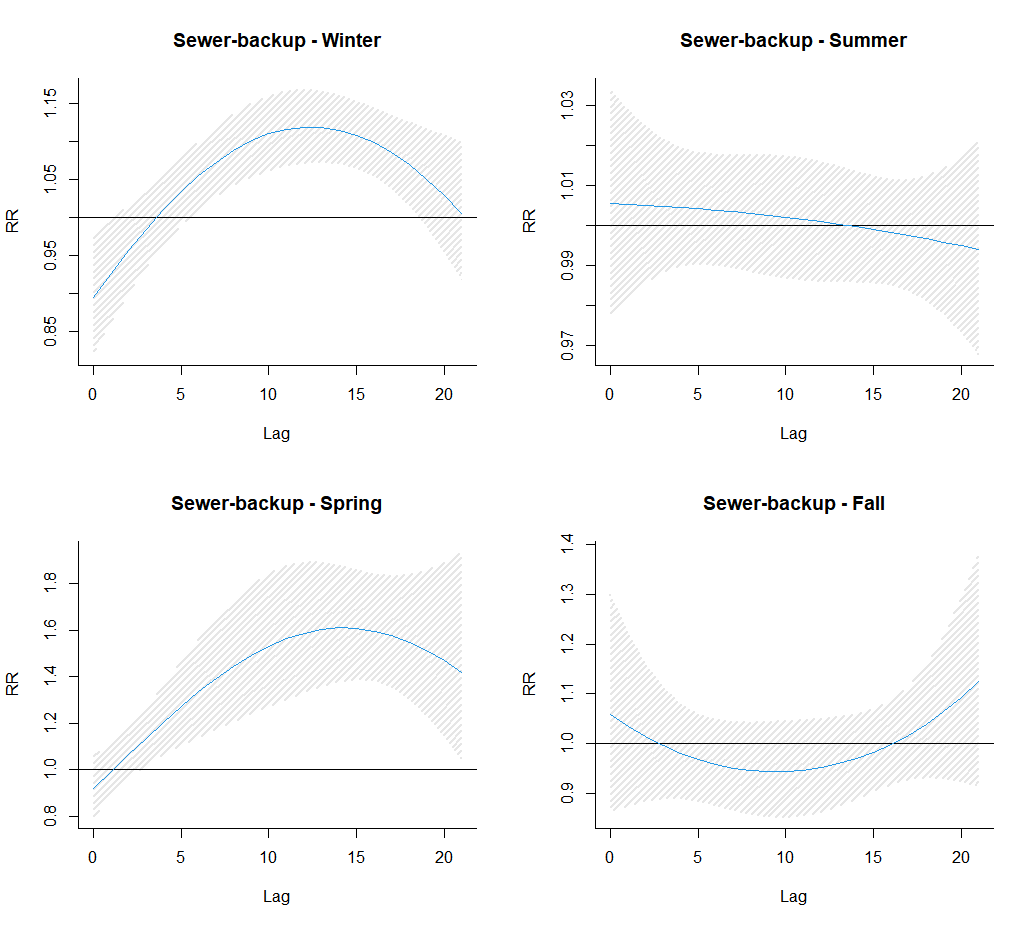 |
| Supplementary Figure S4: Graphical presentation of the association between events of water discoloration, events of sewer-backup and AGI incidence in Toronto across a 21-day lag (secondary analysis) |

| 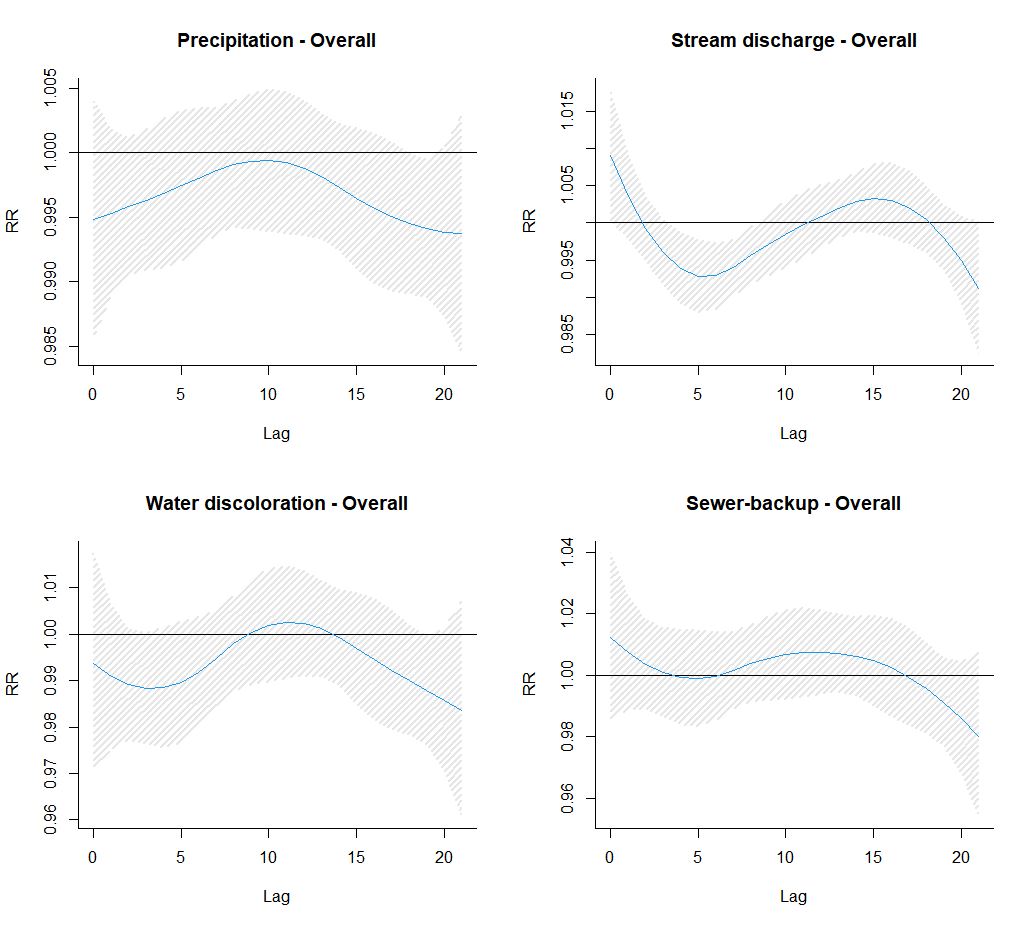 |
| --- |
| 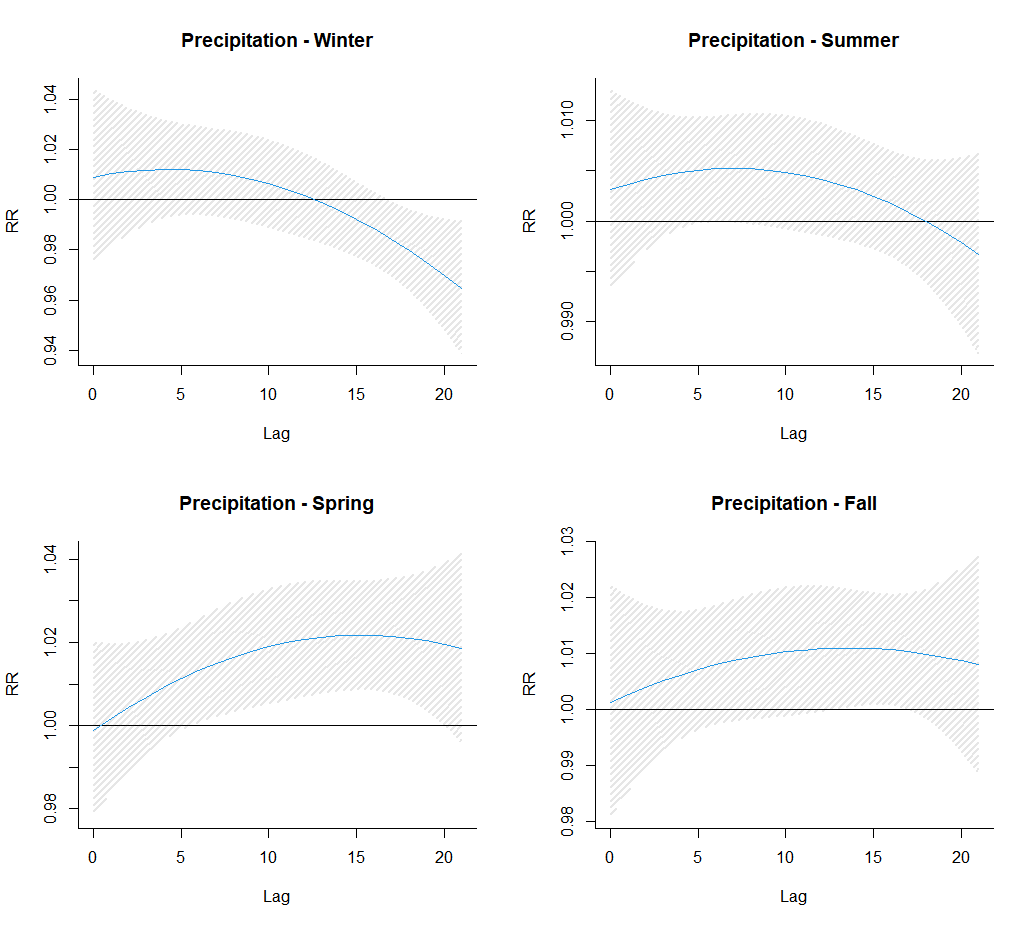 |
| 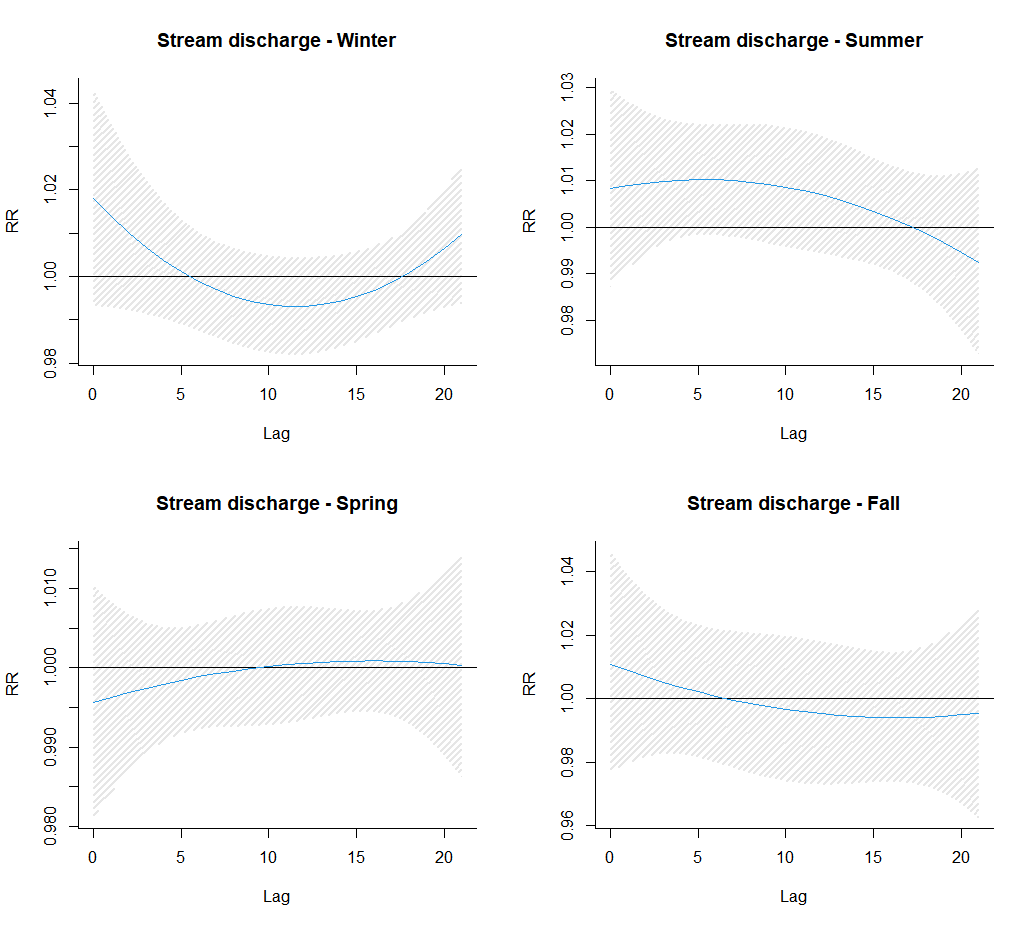 |
| 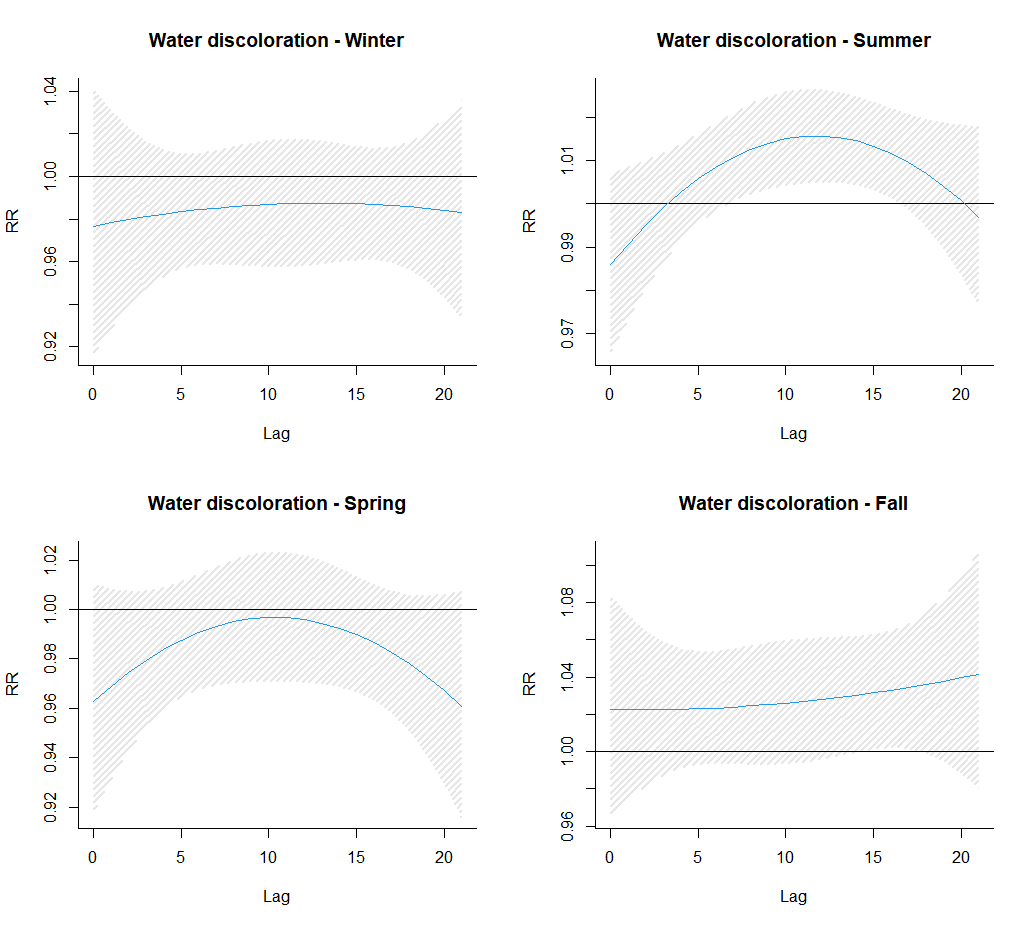 |
| 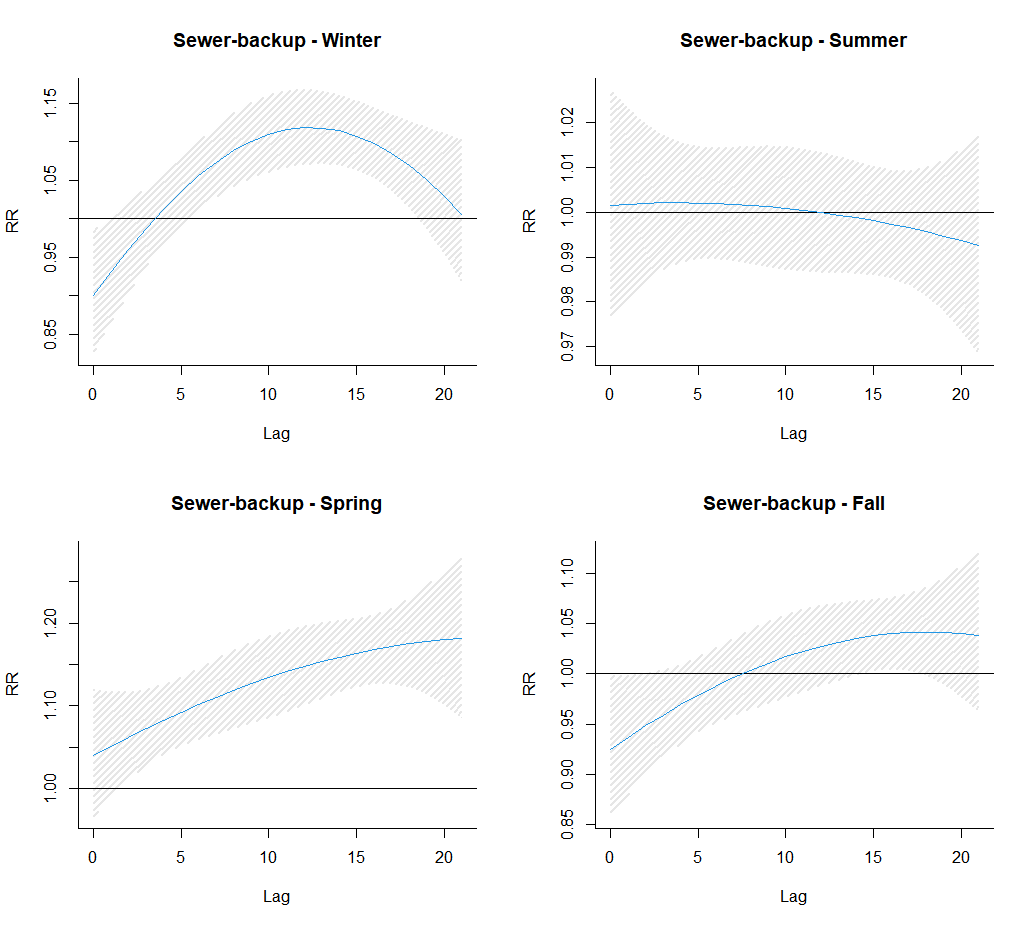 |
| Figure S5: Graphical presentation of the association between extreme precipitation, stream discharge, water discoloration and sewer-backup and AGI incidence in Toronto across a 21-day lag (sensitivity analysis) |
